# Comprehensive Bioinformatics Analysis Reveals PTPN1 (PTP1B) Is a Promising Immunotherapy Target Associated with T Cell Function for Liver Cancer

**DOI:** 10.1155/2023/1533794

**Published:** 2023-01-27

**Authors:** Yihao Zhu, Yao Zu

**Affiliations:** ^1^International Research Center for Marine Biosciences, Ministry of Science and Technology, Shanghai Ocean University, Shanghai 201306, China; ^2^Key Laboratory of Exploration and Utilization of Aquatic Genetic Resources, Ministry of Education, Shanghai Ocean University, Shanghai 201306, China

## Abstract

Recently, PTP1B was identified as a novel immune checkpoint whose removal can unleash T cell responses. However, research on the influence of PTP1B as an immune regulator on liver cancer is limited. This study aimed to investigate the immunological correlation and function of PTP1B in liver cancer. The expression profiles and corresponding clinical information of liver cancer patients were obtained from the TCGA and ICGC databases. GSE146115 and GSE98638 retrieved from the GEO database were used for the single-cell RNA-seq analysis. The mRNA expression of PTP1B (PTPN1) was increased in patients with most malignancies (all *p* < 0.05), including liver cancer (*p* < 0.001). Furthermore, up-regulated PTPN1 was connected to advanced tumor stage (*p* < 0.05) and worse prognosis (*p* < 0.01) in liver cancer. Through Cox regression analysis, PTPN1 was considered as an independent prognosis factor of overall survival (*p* < 0.05) and acted as a high-risk factor (hazard ratio > 1). Gene function and pathway analysis suggested PTPN1 was involved in T cell-related immune responses. Moreover, a close relationship was also found between PTPN1 expression and immune checkpoints as well as immune cells, especially with T cell-related checkpoints (all *p* < 0.001) and T cells (all *p* < 0.001). Single-cell RNA-seq analysis further illustrated that the enrichment of PTPN1 in the T cell population may be linked to its exhaustion in the liver cancer microenvironment. Overall, PTPN1 (PTP1B) closely related to T cell may function as an immunotherapy target for liver cancer.

## 1. Introduction

According to the Global Cancer Statistics of 2020, liver cancer became the seventh most common type of cancer worldwide and the third leading cause of cancer-related death [1]. Clinically, most patients with liver cancer are diagnosed at an advanced stage because the symptoms at an early stage are absent or concealed [2]. In the past decades, although we have made remarkable achievements in improving treatment for liver cancer, the therapy for patients with advanced liver cancer is still limited and ineffective [3]. For patients with advanced liver cancer, systemic chemotherapy is currently the only available and effective treatment [3, 4]. Unfortunately, a large proportion of patients with advanced liver cancer suffer from severe liver dysfunction, often showing poor treatment with systemic chemotherapeutics due to drug resistance [3].

Numerous research studies have demonstrated the complex immunosuppressive network and tumor microenvironment in liver cancer, indicating a promising prospect of immunotherapy, including immune checkpoint therapy and chimeric antigen receptor (CAR) T cell therapy [2, 5]. As an emerging therapy, immunotherapy has been proven to significantly prolong the survival time of patients with liver cancer and improve their prognosis [6]. Notably, the combination treatment of tyrosine kinase inhibitors (TKIs) and anti-PD-1 antibodies proved to be a feasible conversion therapy for patients with advanced or unresectable liver cancer [7]. Nevertheless, there still exist some limitations to immune checkpoint therapy for liver cancer, mainly because of the resistance to immune checkpoint blockades, which is partly related to T cell infiltration defects [2, 8]. Furthermore, CAR‐T therapy for the treatment of liver cancer remains inadequate [9]. Thus, searching for new immune checkpoints closely associated with T cell function may be helpful in improving the situation.

In humans, protein-tyrosine phosphatase 1B (PTP1B), encoded by protein tyrosine phosphatase nonreceptor type 1 (PTPN1), is known to act as a distinct regulatory role in various diseases, such as neurodegenerative diseases, liver disease, cancer, and diabetes [10]. Recently, PTP1B was identified as a novel intracellular immune checkpoint that limits the anti-tumor function of T cells [11]. This leading research also revealed that targeting PTP1B is significant for promoting CAR T cell-mediated antitumor immunity [11]. Accordingly, PTP1B is expected to be a promising target for cancer immunotherapy. However, little is known about the influence of PTP1B on patients with liver cancer [12]. Therefore, elucidating the immunological relevance and function of PTP1B in liver cancer are urgently needed.

In this study, we conducted a comprehensive bioinformatics analysis on PTPN1 (mRNA expression level of PTP1B) in liver cancer based on multiple public databases. A brief flow chart of our research is shown in Supplement Figure S1. Firstly, we investigated the expression profile of PTPN1 at pancancer level via The Cancer Genome Atlas (TCGA) database and Clinical Proteomic Tumor Analysis Consortium (CPTAC) database, as well as the differential expression of PTPN1 in liver cancer from the International Cancer Genome Consortium (ICGC) database and the Human Protein Atlas (HPA) database. Then, we explored the association between the expression of PTPN1 and the clinical characteristics and survival of patients with liver cancer in the TCGA and ICGC databases. To explore whether the biological function and signal pathway of PTPN1 is related to immune responses, three functional and pathway enrichment methods were conducted [13–15]. These methods were the Gene Ontology (GO) and Kyoto Encyclopedia of Genes and Genomes (KEGG) analysis on differentially expressed genes (DEGs) between low- and high-PTPN1 expression groups, the Gene Set Enrichment Analysis (GSEA), and the GO and KEGG analysis on PTPN1-correlated genes. Herein, various immune-related enriched GO terms and KEGG pathways of PTPN1 were observed in the TCGA and ICGC databases. Thus, we furthermore analyzed the correlation of PTPN1 expression with immune checkpoints and tumor infiltrated immune cells, respectively. Using GEO database, single-cell RNA-seq analysis was then performed to identify the PTPN1-expressed immune infiltration cell in liver cancer. Overall, our study shed light on the upregulation of PTPN1 with advanced stage and poor prognosis in liver cancer and revealed PTPN1 (PTP1B) as a potential immunotherapy target due to its close association with immune-related pathways, immune checkpoints, and infiltrating T cells.

## 2. Materials and Methods

### 2.1. Data Resource and Preprocess

The expression profiles (TPM) and corresponding clinical data of liver cancer were downloaded from the TCGA database using the R package “TCGA biolinks,” including 50 normal samples and 374 liver cancer samples. We also downloaded the expression profiles (normalized counts) and clinical data of liver cancer from the LIRI-JP project of the ICGC database (https://dcc.icgc.org/releases/current/Projects/LIRI-JP), containing 202 normal samples and 243 liver cancer samples, as the validation dataset for our study. Samples with missing or uncertain information in these datasets were excluded. These samples were then divided into low- and high-PTPN1 expression groups according to the median expression of PTPN1. Moreover, GSE146115 and GSE98638 were obtained from the GEO database for the single-cell RNA-seq analysis [16, 17].

### 2.2. Analysis of PTPN1 Expression in Pancancer and Liver Cancer

The TIMER (https://cistrome.shinyapps.io/timer/) and UALCAN (https://ualcan.path.uab.edu) online tools were used to analyze the differential expression of PTPN1 in distinct cancer types and liver cancer from the TCGA database and the CPTAC database, respectively [18, 19]. The expression of PTPN1 in liver cancer was then analyzed in the ICGC database by the R package “ggpubr.” The HPA database (https://www.proteinatlas.org/) was further utilized to study the protein expression levels of PTPN1 in liver cancer and normal liver. In our study, the expression of PTPN1 in the TCGA database was normalized as log_2_ (TPM), which is consistent with the method that TIMER used. Furthermore, *Z*-value and log_2_ (normalized counts) were separately applied to measure the expression of PTPN1 in the CPTAC database and the ICGC database.

### 2.3. Correlation Analysis of Clinical Characteristics

To explore the correlation between PTPN1 expression and distinct clinical characteristics of liver cancer, the normal samples were excluded, and clinical characteristics (gender, tumor stage, and Tumor Node Metastasis classification (TNM classification)) were converted into numerical variables for further analysis. The R package “ggplot2” and “ComplexHeatmap” were used to describe the distribution of survival status and clinical characteristics of patients in the TCGA database and the ICGC database according to the increased expression of PTPN1. Then, the expression of PTPN1 among different groups based on age, gender, tumor grade, and TNM classification was investigated using the R package “ggpubr.”

### 2.4. Survival Analysis

To identify the prognostic value of PTPN1 in liver cancer, tumor samples were classified into low- and high-PTPN1 expression groups according to the best cutoff value of PTPN1 using the function “surv_cutpoint” inserted in the R package “survminer.” Next, K-M curves were plotted to illustrate the relationship of PTPN1 expression and overall survival (OS) using the R package “survminer” and “survival.” To test whether PTPN1 was an independent prognostic factor of OS, univariate and multivariate Cox regression analyses were performed using the R package “survival.”

### 2.5. Gene Function and Pathway Analysis by GO, KEGG, and GSEA

To investigate the potential biological function and pathway, we first screened the DEGs between the low- and high-PTPN1 expression groups using the R package “limma.” The genes with log_2_|fold change (FC)| larger than one and adjusted *p* value (*q* value) lower than 0.05 were considered significant DEGs [14]. Next, these DEGs were used to perform GO and KEGG pathway enrichment analysis. GO terms and KEGG pathways with *q* value lower than 0.05 were significantly enriched in DEGs [14]. Furthermore, we also utilized GSEA pathway analysis on PTPN1 by the KEGG gene sets. GO, KEGG, and GSEA analysis were conducted by the R package “clusterProfiler.”

### 2.6. PTPN1-Related Genes Enrichment Analysis by GO and KEGG

Considering the limitations of GO and KEGG analysis based on the DEGs, the method using correlated genes was utilized to annotate the function and pathways of PTPN1 for verification. Firstly, the GeneMANI database (https://genemania.org/) and STRING database (https://cn.string-db.org/) were used to display PTPN1-dominated gene interaction network and protein interaction network, respectively. Next, the interactive genes with PTPN1 from GeneMANI database and STRING database were analyzed by Pearson's correlation to obtain the significant part, combined with 60 genes encoding immune regulators (Supplement Table 1) [20, 21]. Moreover, we used Pearson's correlation method to screen out the top 100 PTPN1-related genes in the TCGA database and ICGC database, respectively, and the top 10 significant correlated genes in the TCGA and ICGG databases were then visualized as a chordal graph by the R package “circlize.” Accordingly, the important genes positively related with PTPN1 (*p* < 0.001 and Pearson *R* > 0) were obtained (Supplement Table 2), which were used for GO and KEGG pathway analysis in the TCGA and IGCG databases. GO terms and KEGG pathways with *q* value lower than 0.05 were considered the significantly enrichment of PTPN1-correlated genes. GO and KEGG analyses were performed by the R package “clusterProfiler.”

### 2.7. Correlation Analysis of Immune Checkpoints

To explore whether the expression of PTPN1 was correlated with immune checkpoints in liver cancer, 47 members of immune checkpoints were obtained for our study [20]. Using the R package “ggpubr,” the expression profiles of these immune checkpoints among low- and high-PTPN1 expression groups in the TCGA database and ICGC database were revealed. Next, we assessed and visualized the significant correlations between the immune checkpoints and PTPN1 expression by the R package “corrplot.” The top 10 important immune checkpoints correlated with PTPN1 in the TCGA database and ICGC database were displayed in chordal graph by the R package “circlize,” respectively.

### 2.8. Correlation Analysis of Immune Infiltration Cells

To illustrate the relationship between PTPN1 and immune infiltration cells in liver cancer, the TIMER online tool was adopted to analyze. Herein, the relationship between PTPN1 and immune infiltration cells was investigated from the infiltration abundances and immune gene expressions, respectively. TIMER applied the constrained least squares method to estimate the infiltration abundance of six immune cells in liver cancer from the TCGA database [18]. Firstly, we evaluated the relationship between PTPN1 expression and infiltration levels of six immune cells (B cell, CD8+ T cell, CD4+ T cell, macrophage, neutrophil, and dendritic cell) using Spearman's correlation method. To further explore more specific types of immune cells, the gene markers of B cell (CD19, CD79A), T cell (CD3D, CD3E, CD3G), CD4+ T cell (CD4), CD8+ T cell (CD8A, CD8B), Treg cell (IL2RA, CTLA), Th cell (CCL5, GZMA), macrophage (CD68, CD163), dendritic cell (CD1C, ITGAX), and NK cell (KLRF1, KLRD1) were also included for Spearman's correlation analysis. The gene markers of immune cells in liver cancer were obtained from Cell Marker 2.0 database (https://yikedaxue.slwshop.cn/) [22].

### 2.9. Single-Cell RNA-Seq Analysis

To precisely identify the PTPN1-expressed immune cells infiltrated in liver cancer, we conducted single-cell RNA-seq analysis on GSE146115 obtained from the GEO database. The R package “Seurat” was used for clustering analysis based on PCA and UMAP algorithms. Then, we utilized the R package “SingleR” to identify the cell types of different clusters. In order to annotate cells more accurately, the marker genes of liver cancer cells and immune cells from the Cell Marker 2.0 database were obtained to correct the wrongly identified cell types. Next, we further identified the T cell subtypes in GSE146115 and then utilized the R package “Monocle” for trajectory analysis using the “DDRTree” method. The additional processes of single-cell RNA-seq analysis on GSE146115 are shown in Supplement Figures S2 and S3. Furthermore, we analyzed GSE98638 dataset using TISCH online tool (https://tisch.comp-genomics.org/) to verify the results of GSE146115 [23].

### 2.10. Statistical Analysis

Statistical analysis was performed using R programming language (version 4.2.0). Wilcoxon test or unpaired *t*-test was applied to evaluate the expression of PTPN1 in tumor tissues versus normal tissues. Unpaired *t*-test was also utilized to investigate the relationship between PTPN1 expression and various clinical characteristics (all converted to numerical variables) of liver cancer. Log rank test was used to analyze the difference of OS between low- and high-PTPN1 expression groups. Pearson's correlation method was conducted to screen the correlated genes of PTPN1, and the correlation between PTPN1 expression and immune checkpoints was evaluated. Spearman's correlation analysis was performed to test the correlation of PTPN1 expression between infiltration levels and gene markers of immune cells. The statistical analyses were all two-tailed tests, and *p* < 0.05 was considered statistically significant.

## 3. Results

### 3.1. Expression Profile of PTPN1 at Pancancer Level

**Figure 1:**
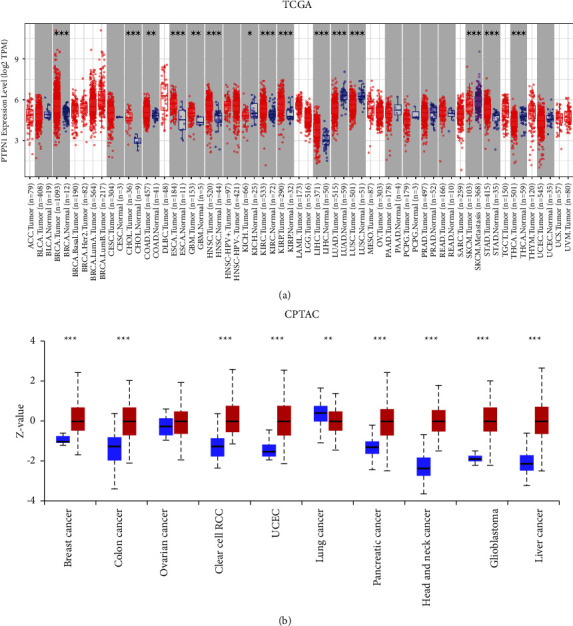
Expression of PTPN1 in different types of cancers. PTPN1 expression levels in different cancers from the TGCA database (a) and the CPTAC database (b). ^*∗*^*p* < 0.05, ^*∗∗*^*p* < 0.01, and ^*∗∗∗*^*p* < 0.001.

First, the expression of PTPN1 in different types of cancer was analyzed by the TIMER online tool. The box plots showed that PTPN1 was upregulated in breast invasive carcinoma (BRCA), cholangiocarcinoma (CHOL), colon adenocarcinoma (COAD), esophageal carcinoma (ESCA), glioblastoma multiforme (GBM), head and neck squamous cell carcinoma (HNSC), kidney chromophobe (KICH), kidney renal clear cell carcinoma (KIRC), kidney renal papillary cell carcinoma (KIRP), liver hepatocellular carcinoma (LIHC), lung adenocarcinoma (LUAD), lung squamous cell carcinoma (LUSC), skin cutaneous melanoma (SKCM), stomach adenocarcinoma (STAD), and thyroid carcinoma (THCA) compared with matched normal tissues (all *p* < 0.05, Figure 1(a)). Then, the UALCAN online tool was utilized to validate the result. As shown in Figure 1(b), high expression of PTPN1 was also observed in breast invasive carcinoma, colon adenocarcinoma, lung squamous cell carcinoma, head and neck squamous cell carcinoma, glioblastoma multiforme, and liver cancer (all *p* < 0.01). The data showed that PTPN1 was overexpressed in distinct types of cancers, indicating its potential carcinogenic role.

### 3.2. PTPN1 Is Up-regulated in Liver Cancer

**Figure 2:**
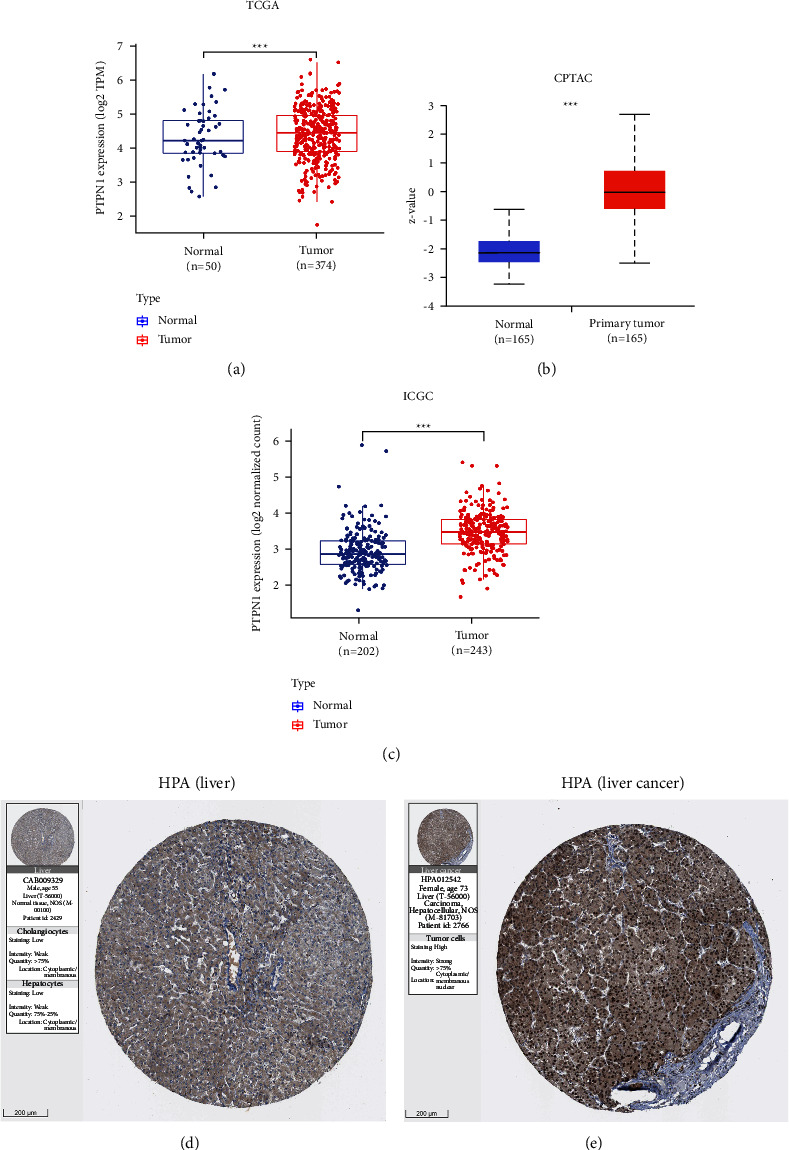
Expression distribution of PTPN1 in liver cancer tissues and normal tissues. Increased expression of PTPN1 in liver cancer in the TCGA database (a), CPTAC database (b), ICGC database (c). Protein expression level of PTPN1 in normal liver (d) and liver cancer (e). ^*∗∗∗*^*p* < 0.001.

The increased expression of PTPN1 in liver cancer was confirmed by the ICGC database. The result of the ICGC database (*p* < 0.001, Figure 2(c)) was consistent with the TCGA database (*p* < 0.001, Figure 2(a)) and CPTAC database (*p* < 0.001, Figure 2(b)). We further assessed the protein expression level of PTPN1 in liver cancer using the HPA database. Immunohistochemical staining showed that PTPN1 was significantly upregulated in liver cancer tissue compared to normal liver tissue (Figures 2(d) and 2(e)).

### 3.3. Association between PTPN1 Expression and Clinical Characteristics of Liver Cancer

**Figure 3:**
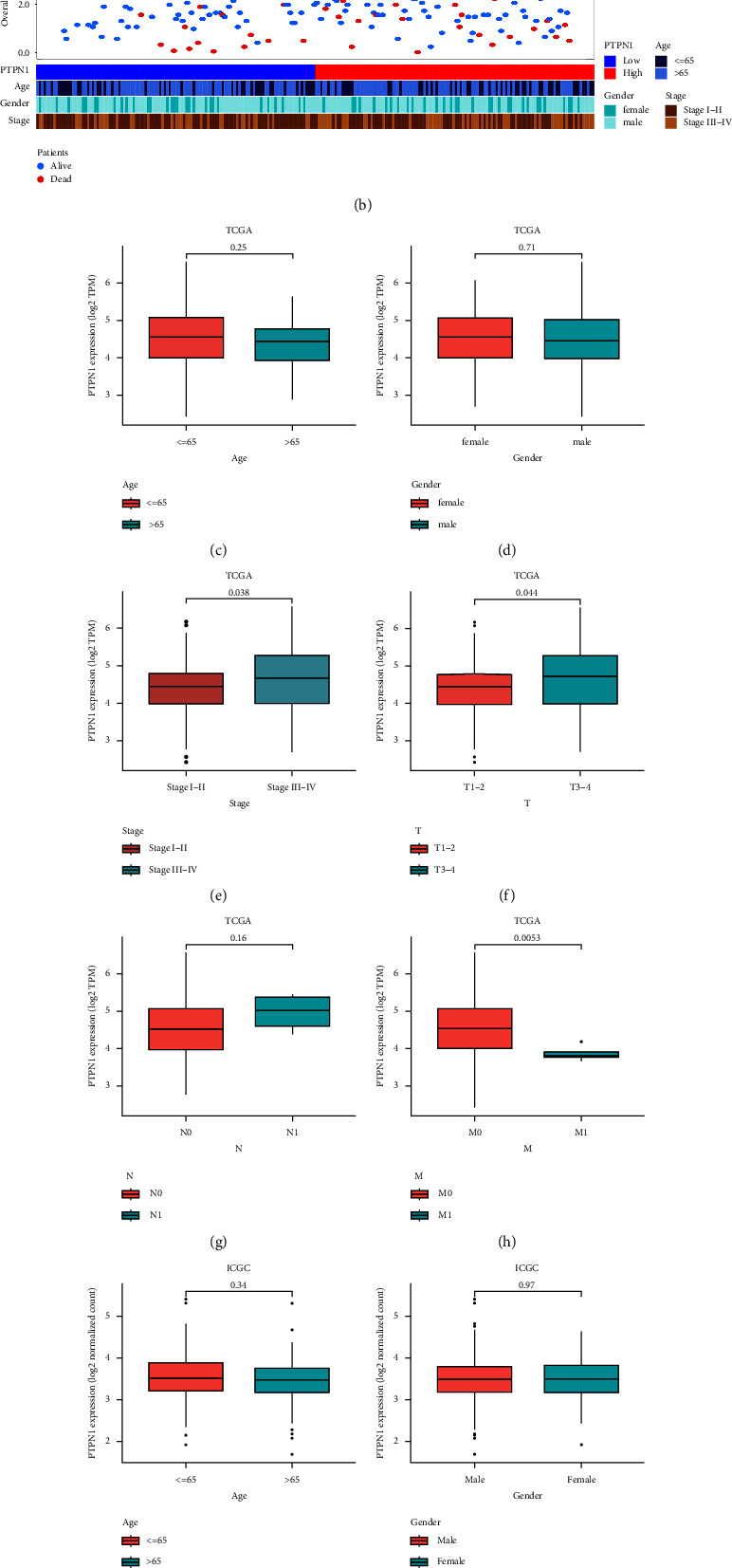
PTPN1 expression among different groups of liver cancer patients based on clinical features. The landscapes of survival status and clinical characteristics of patients with liver cancer in the TCGA database (a) and ICGC database (b). Boxplots show the association between PTPN1 expression and age (c), gender (d), cancer stage (e), TNM classification (f, g, h) in the TCGA database. The association between PTPN1 expression and age (i), gender (j), and cancer stage (k) in the ICGC database.

To explore the relationship between PTPN1 and clinical characteristics of liver cancer, scatter plots and heatmaps were first utilized to show the distribution of survival status and clinical features of liver cancer patients in the TCGA database (Figure 3(a)) and the ICGC database (Figure 3(b)). The results indicated that the higher expression of PTPN1 was linked with the accumulation of dead patients and patients with advanced stage (stage III-IV). We then compared the PTPN1 expression in distinct patients based on clinical characteristics. The TCGA and ICGC databases demonstrated that the expression of PTPN1 did not correlate with patients' age (*p* > 0.05, Figures 3(c) and 3(i)) or gender (*p* > 0.05, Figures 3(d) and 3(j)). Patients with stage III-IV showed higher PTPN1 expression in the TCGA database (*p* < 0.05, Figure 3(e)) and the ICGC database (*p* < 0.05, Figure 3(k)). Moreover, PTPN1 expression was correlated with the T stage (*p* < 0.05, Figure 3(f)) and M stage (*p* < 0.001, Figure 3(h)) but not with the N stage (*p* > 0.05, Figure 3(g)). These results suggested that PTPN1 was upregulated in patients with advanced stage, indicating its important role in the development of liver cancer.

### 3.4. Prognostic Value of PTPN1 in Liver Cancer

**Figure 4:**
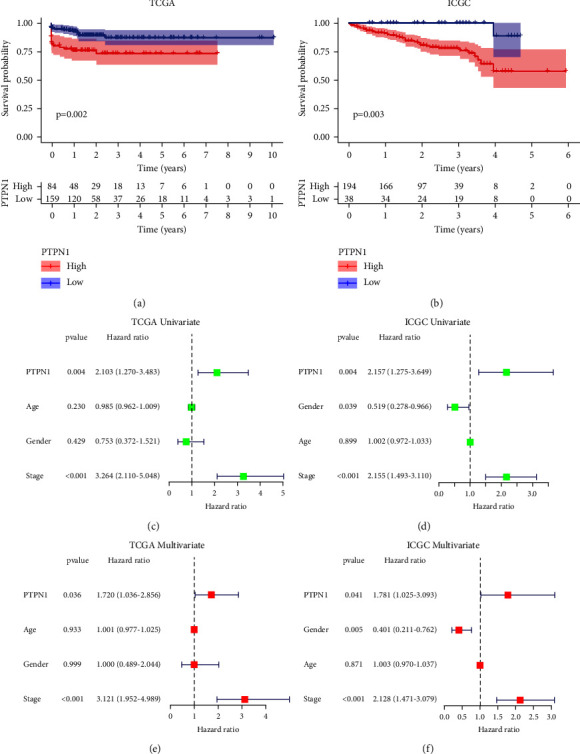
Prognostic value of PTPN1 in liver cancer. Kaplan–Meier analysis of OS between high- and low-PTPN1 expression groups in the TCGA database (a), ICGC database (b), univariate Cox regression analysis of PTPN1 expression and clinical characteristics in the TCGA database (c), ICGC database (d), multivariate Cox regression analysis of PTPN1 expression and clinical characteristics in the TCGA database (e), and ICGC database (f).

To evaluate the prognostic value of PTPN1 in liver cancer, Kaplan–Meier analysis and Cox regression analysis were conducted. The Kaplan–Meier curve for OS showed that patients with higher expression of PTPN1 had a worse prognosis in the TCGA database (*p*=0.002, Figure 4(a)) and the ICGC database (*p*=0.003, Figure 4(b)). Univariate and multivariate Cox regression analysis was then used to confirm whether PTPN1 was an independent prognostic factor of liver cancer. The results indicated that PTPN1 was independent of clinical features (including age, gender, and stage) and acted as a high-risk factor (hazard ratio > 1) for liver cancer patients in the TCGA database (Figures 4(c) and 4(e)) and the ICGC database (Figures 4(d) and 4(f)).

### 3.5. PTPN1 Is Involved in Immune-Related and Cell Adhesion-Related Signaling Pathways

**Figure 5:**
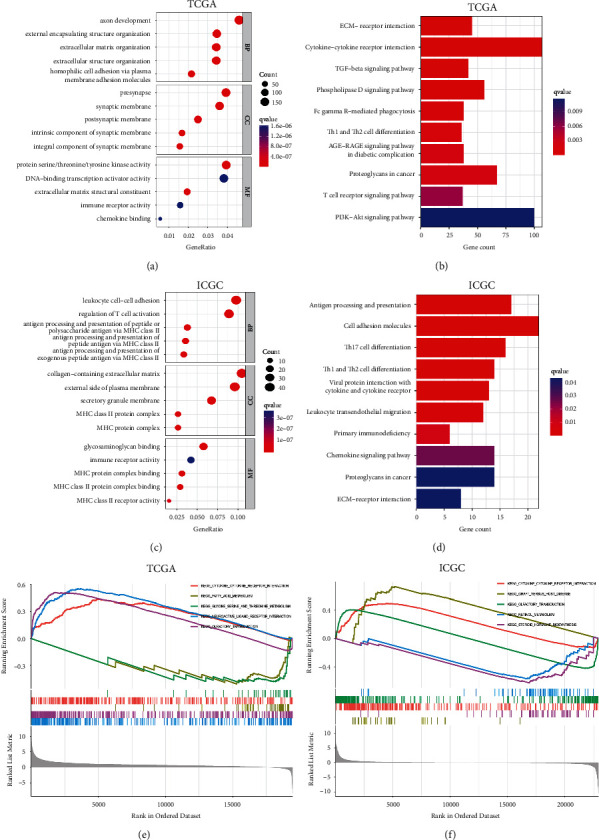
GO, KEGG, and GSEA pathway analysis for PTPN1. (a) Enrichment GO terms of DEGs in the TCGA database, (b) enrichment KEGG pathways of DEGs in the TCGA database, (c) enrichment GO terms of DEGs in the ICGC database, and (d) enrichment KEGG pathways of DEGs in the ICGC database. GSEA analysis of PTPN1 using KEGG gene sets in the TCGA database (e) and ICGC database (f).

To illustrate the potential biological functions and pathways of PTPN1 in liver cancer, we first screened DEGs between the high-expression group and the low-expression group of PTPN1. Then, GO and KEGG analyses were performed based on these DEGs. In the TCGA database, the enriched GO terms (*q* < 0.05, Figure 5(a)) and KEGG pathways (*q* < 0.05, Figure 5(b)) were closely associated with immune response receptors and cell adhesion, including immune receptor activity, chemokine binding, cytokine-cytokine receptor interaction, T cell receptor signaling pathway, ECM-receptor interaction, and proteoglycans in cancer. Then, GO and KEGG analyses were utilized in the ICGC database to validate the above findings. The results of the ICGC database also showed that the enriched GO terms (*q* < 0.05, Figure 5(c)) and KEGG pathways (*q* < 0.05, Figure 5(d)) were related to immune response and cell adhesion. To further identify the specific molecular mechanism affected by PTPN1 in liver cancer, we also conducted a GSEA pathway analysis using KEGG gene sets. Interestingly, cytokine-cytokine receptor interaction was also found and significantly enriched in the up-regulated groups of PTPN1 in the TCGA database (*p* < 0.05, Figure 5(e)) and the ICGC database (*p* < 0.05, Figure 5(f)). Therefore, PTPN1 may be involved in immune responses of liver cancer, in which the cytokine-cytokine receptor interaction pathway may be the underlying mechanism.

### 3.6. PTPN1 Regulates Immune Response Processes with Its Interactive Genes

**Figure 6:**
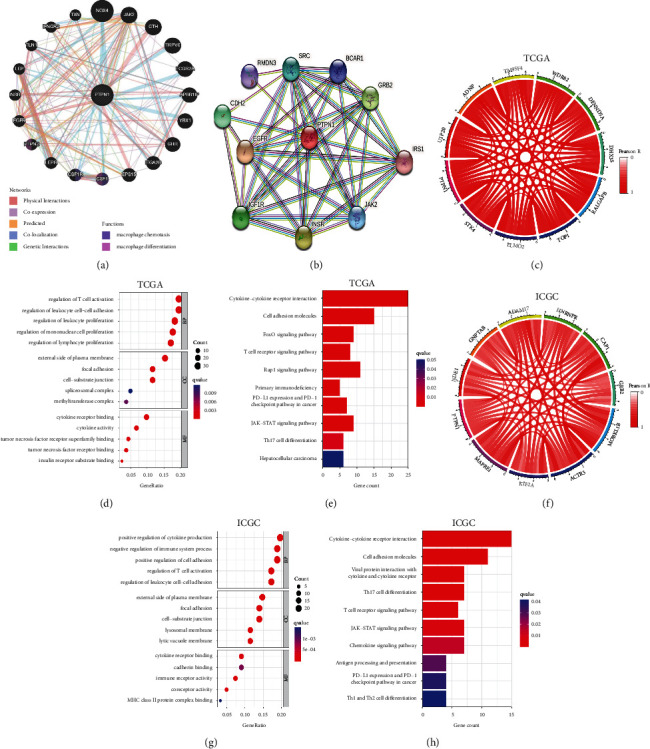
Interaction networks and pathway analysis of PTPN1-related genes. (a) Gene interaction network of PTPN1 using the GeneMANI database. (b) PTPN1-dominated protein interaction network constructed by the STRING database. (c) Top 10 genes closely correlated with PTPN1 in the TCGA database. (d) Enrichment GO terms of PTPN1-related genes in the TCGA database. (e) Enrichment KEGG pathways of PTPN1-related genes in the TCGA database. (f) Top 10 genes closely correlated with PTPN1 in the ICGC database. (g) Enrichment GO terms of PTPN1-related genes in the ICGC database. (h) Enrichment KEGG pathways of PTPN1-related genes in the ICGC database.

To ensure the accuracy of the above results, GO and KEGG analyses based on PTPN1-related genes were performed in the TCGA database and the ICGC database as another strategy to annotate biological functions and pathways. As shown in Figures 6(a) and 6(b), the GeneMANI database and STRING database provided us with the gene-gene and protein-protein interaction network of PTPN1, respectively. Based on these networks, 27 genes interacting with PTPN1 were obtained for further analysis (duplicate genes are excluded, see Supplement Table 1). Furthermore, the top 100 PTPN1-correlated genes and 60 genes encoding immune regulators are also included in our analysis (Supplement Table 1). Pearson correlation analysis was then used to screen out the significantly correlated genes with PTPN1 (*p* < 0.001). Herein, we ultimately obtained a set of 165 genes and a set of 138 genes in the TCGA database and the ICGC database, respectively (Supplement Table 2). The top 10 genes positively correlated with PTPN1 in the TCGA database and the ICGC database are shown in Figures 6(c) and 6(f), respectively. In the TCGA database, PTPN1 interacting genes are enriched in many immune response-related functions and pathways (*q* < 0.05, Figures 6(d) and 6(e)), such as regulation of T cell activation, cytokine-cytokine receptor interaction, T cell receptor signaling pathway, and Th17 cell differentiation. These results are then confirmed in the ICGC database (*q* < 0.05, Figures 6(g) and 6(h)). Notably, most of the results pointed to T cell-related immune responses and pathways, indicating that the function of T cells might be affected by PTPN1 and its related genes in liver cancer.

### 3.7. Correlation Analysis between PTPN1 Expression and Immune Checkpoint Members in Liver Cancer

**Figure 7:**
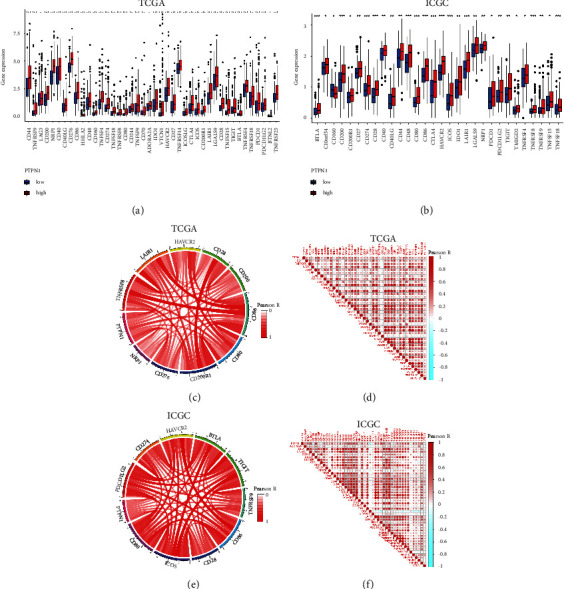
Expression patterns of 47 types of immune checkpoints and their correlations with PTPN1. (a, b) The upregulated expression of immune checkpoints in the TCGA database and the ICGC database. (c) Top 10 immune checkpoints closely correlated with PTPN1 in the TCGA database. (d) Pearson's correlation heatmap of PTPN1 and immune checkpoints in the TCGA database. (e) Top 10 immune checkpoints closely correlated with PTPN1 in the ICGC database. (f) Pearson's correlation heatmap of PTPN1 and immune checkpoints in the ICGC database. ^*∗*^*p* < 0.05, ^*∗∗*^*p* < 0.01, and ^*∗∗∗*^*p* < 0.001.

PTPN1 was proven to show synergistic effects with some immune checkpoints, such as PD-1 [11]. Herein, we analyzed the expression patterns of 47 immune checkpoint members and their correlations with PTPN1. As presented in Figures 7(a) and 7(b), the up-regulated expression profiles of most immune checkpoints are observed in the high-expression groups of PTPN1 (*p* < 0.05). The top 10 immune checkpoints closely correlated with PTPN1 in the TCGA database and ICGC database are shown in Figures 7(c) and 7(e), respectively. Among these immune checkpoints closely interacting with PTPN1 in the TCGA and ICGC databases, CD28, CD80, and CD86 are notable intersections closely related to T cell function. The heatmaps showed the immune checkpoints significantly related to PTPN1 (*p* < 0.001) in the TCGA and ICGC databases (Figures 7(d) and 7(f)), showing the positive correlation between the expression of most immune checkpoints and PTPN1 in liver cancer.

### 3.8. Correlation Analysis between PTPN1 Expression and Immune Cell Infiltration in Liver Cancer

**Figure 8:**
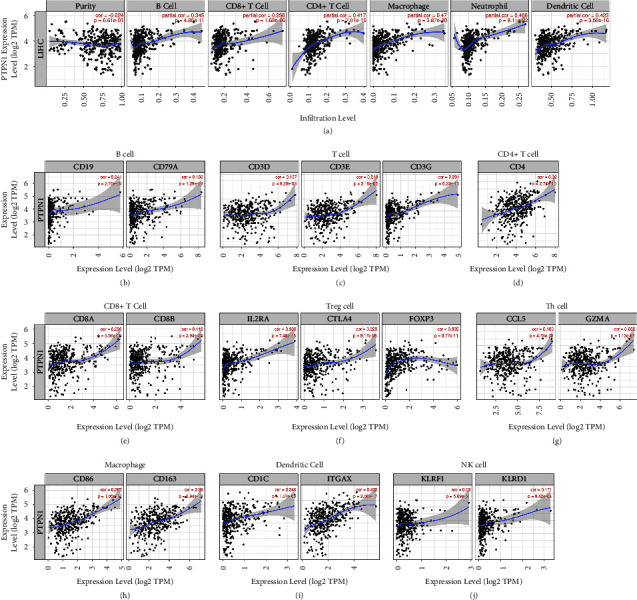
Expression of PTPN1 is related to infiltration levels and gene markers of immune cells. (a) Relationship of PTPN1 expression and the immune cell infiltration. Expression relationship between PTPN1 and the gene markers of B cell (b), T cell (c), CD4+ T cell (d), CD8+ T cell (e), Treg cell (f), Th cell (g), macrophage (h), dendritic cell (i), and NK cell (j) in liver cancer.

To elucidate whether PTPN1 was related to immune infiltration cells in liver cancer, the TIMER database was used to analyze the correlation between the expression of PTPN1 and six types of immune infiltration cells, including B cell, CD8+ T cell, CD4+ T cell, macrophage, neutrophil, and dendritic cell.  Figure 8(a) shows that PTPN1 expression was positively correlated with the infiltration levels of the above six immune cells (all Spearman *R* > 0, all *p* < 0.001). Next, we assessed the expression correlation between PTPN1 and gene markers of nine immune cells. The expression of PTPN1 was closely positive (most Spearman *R* > 0.2) associated with B cell (Figure 8(b)), T cell (Figure 8(c)), CD4+ T cell (Figure 8(d)), CD8+ T cell (Figure 8(e)), Treg cell (Figure 8(f)), macrophage (Figure 8(h)), and dendritic cell (Figure 8(i)) but weakly positive (all Spearman *R* < 0.2) associated with Th cell (Figure 8(g)) and NK cell (Figure 8(j)). These results indicated a close linkage between PTPN1 and most immune cells in liver cancer, in which T cell might be an essential cell type affected by PTPN1.

### 3.9. Distribution of PTPN1 in Different Subtypes of T Cells

**Figure 9:**
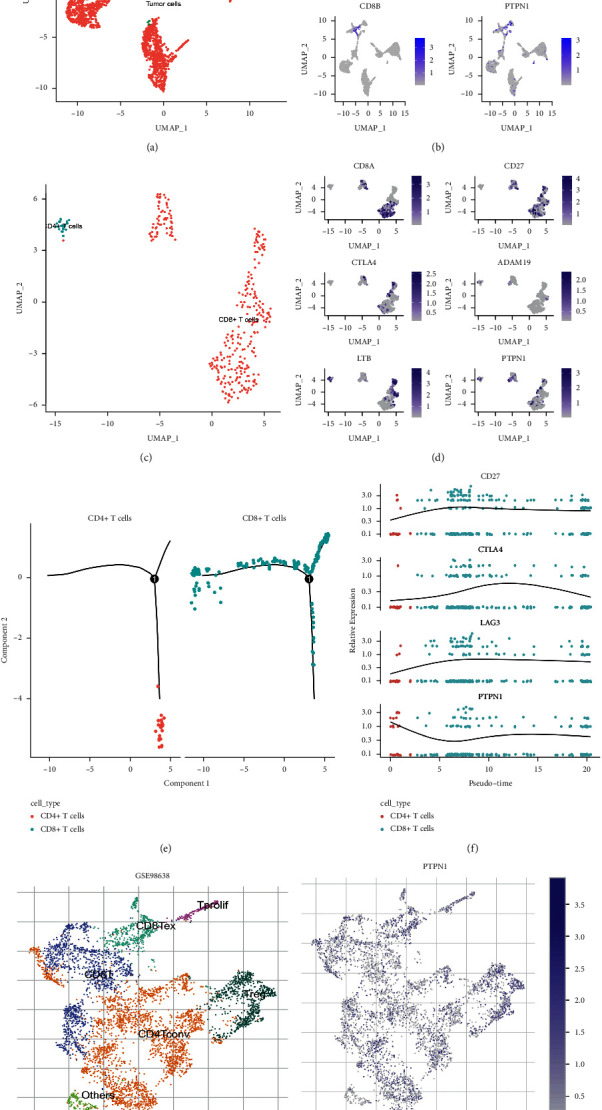
Identification of PTPN1-expressed immune cells based on single-cell RNA-seq analysis. (a) Tumor cells and immune cells in GSE146115. (b) Distribution of T cell markers and PTPN1 in different cell types in GSE146115. (c) Subtypes of T cells in GSE146115. (d) Distribution of T cell markers and PTPN1 in T cell subtypes. (e) Monocle 2 trajectory of T cell population in GSE146115. (f) The expression of T cell exhaustion-related genes and PTPN1 in T cells ranked by pseudotime state in GSE146115. (g, h) The immune cell types and PTPN1 distribution in GSE98638.

To further explore the PTPN1-expressed immune cells in liver cancer, two datasets (GSE146115 and GSE98638) were obtained for the single-cell RNA-seq analysis. The entire cell profile of the GSE146115 dataset was projected onto the UMAP plot (Figure 9(a)). Using the R package “singleR” and specific cell markers, five main types of cells were annotated in the GSE146115 dataset, including tumor cells, B cells, T cells, macrophages, and NK cells. We then explore the expression of PTPN1 and gene markers of two classical T cell subtypes (CD4+ and CD8+ T cell) in these cells. As can be seen from Figure 9(b), CD4, CD8A, and CD8B were all expressed in the T cell population, and PTPN1 was also enriched. Considering that the PTPN1 expression may be influenced by the heterogeneity of the T cell population, we further identified the specific subtype of T cells in GSE146115. As shown in Figures 9(c) and 9(d), the T cells were divided into CD4+ and CD8+ subtypes according to the gene markers.  Figure 9(d) further demonstrated that CD4+ and CD8+ T cells were all PTPN1-expressed cell types while exhibiting the maker genes of T cells infiltrating liver cancer.

Moreover, Monocle 2 trajectory analysis was performed to distinguish the T cell population based on the pseudotime state. The T cell population is classified into three distinct states as shown in Figure 9(e).  Figure 9(f) further demonstrated the expression linkage pattern of PTPN1 and T cell exhaustion-related genes (CD27, CTLA4, and LAG3) in CD4+ and CD8+ T cells, and had minor relationships with the cell states. GSE98638 dataset was then analyzed by TISCH online tool to verify the above results. The profile of T cells infiltrated in liver cancer from the GSE98638 dataset is releveled in Figure 9(g), including conventional CD4+ T cells (CD4Tconv), CD8+ T cells (CD8T), regulatory T cells (Treg), exhausted CD8+ T cells (CD8Tex), proliferating T cells (Tprolif), and other cells. Significantly, the overexpression distribution pattern of PTPN1 was observed in the five distinct types of T cells from GSE98638 (Figure 9(h)). Overall, the data suggested that PTPN1 was enriched in different types of T cells in GSE146115 and GSE98638, some of which were exhausted cells or displayed exhaustion characteristics; thus, indicating the potential relationship between PTPN1 expression and exhausted T cells infiltrated in liver cancer.

## 4. Discussion

As the founding member of the protein tyrosine phosphatase (PTP) superfamily, PTP1B (encoded by PTPN1) has been reported to exert critical roles in many physiological and pathological processes, such as insulin signal transduction and cancer development [10, 11]. Accordingly, PTP1B has long been regarded as a very attractive therapeutic target against metabolic syndrome and cancer [10, 11]. Recently, a leading study uncovered that PTP1B is an immune checkpoint upregulated in tumor T cells and the inactivation of PTP1B can enhance T cell-mediated antitumor immunity [11]. The research suggested that PTP1B is a promising immunotherapy target, especially for providing an alternative for CAR‐T cell immunotherapy in solid tumors [11]. Nevertheless, our understanding of the influence and function of PTP1B in liver cancer remains limited. Therefore, more attention should be paid to exploring the role of PTP1B in liver cancer.

In this study, the TIMER and UALCAN online tools were first used to investigate the differential mRNA expression of PTP1B (PTPN1) at a pancancer level. We found that PTPN1 was overexpressed in various cancers, including liver cancer. Meanwhile, the upregulation of PTPN1 in liver cancer was validated in the ICGC database and the HPA database, respectively. The results indicated the oncogenic role of PTPN1 in the development of liver cancer. Previous research has shown that PTPN1 increased in liver cancer tissues or liver cancer cell lines [24, 25], which was consistent with our analysis from public databases.

Next, we explore the relationship between PTPN1 expression and clinical characteristics of liver cancer. The results demonstrated that PTPN1 expression was enriched in patients with advanced stage in the TCGA and the ICGC databases, which further illustrated the oncogenic role of PTPN1 in liver cancer. Since PTPN1 was considered a potential oncogenic gene related to patients with advanced liver cancer, we also wonder whether PTPN1 has a prognostic significance for liver cancer. Kaplan–Meier analysis showed that the higher expression of PTPN1 was connected to a shorter prognosis time (OS) of liver cancer patients. Univariate and multivariate Cox regression analysis then revealed PTPN1 as an independent prognostic factor with a high risk of liver cancer. Overall, these results suggested that the up-regulated expression of PTPN1 was associated with advanced tumor stage and poor prognosis of patients with liver cancer.

Inspired by the reported role of PTPN1 as an immune checkpoint [11], gene function and pathway analysis were performed to explore whether PTPN1 was involved in immune response processes in liver cancer. Herein, three strategies were adopted for more credible results. First, the GO and KEGG analyses based on DEGs were conducted to annotate the PTPN1-related biological functions and pathways. The majority of enriched GO terms and KEGG signaling pathways closely related to immune response and cell adhesion were observed in the TCGA database and the ICGC database, which mainly included immune receptor activity, chemokine binding, cytokine-cytokine receptor interaction, T cell receptor signaling pathway, ECM-receptor interaction, and proteoglycans in cancer. However, this traditional analysis using overlap statistics merely focuses on the DEGs' enriched ontology terms or pathways [26]. Compared to traditional GO/KEGG analyses, GSEA comprehensively considered all the genes rather than DEGs determined by certain threshold values [26]. Thus, GSEA as an additional method was performed for functional and pathway analysis. The GSEA results significantly showed that upregulated PTPN1 was involved in the cytokine-cytokine receptor interaction in the TCGA and ICGC databases. Subsequently, we may speculate that the cytokine-cytokine receptor interaction acts as a critical mechanism in PTPN1-regulated immune responses in liver cancer.

To deepen our understanding of the mechanism of PTPN1 in liver cancer and verify the above results, we also carried out GO and KEGG analyses based on PTPN1-correlated genes. The correlation genes from four sources (Supplement Table 1) were adopted for analysis. In the TCGA and ICGC databases, PTPN1 interactive genes were observed participating in many T cell-related immune functions and pathways, such as regulation of T cell activation, cytokine-cytokine receptor interaction, and T cell receptor signaling pathway. Interestingly, the enrichment results also included the cytokine-cytokine receptor interaction, consistent with our present analysis. Together, we found strong evidence that PTPN1 may function as an immune regulator that affects the functions of T cells infiltrated in liver cancer. The closely relevant pathway was the cytokine-cytokine receptor interaction. Numerous research have underscored the linkage between cytokine and T cell function [27, 28]. Impressively, Wiede et al. illustrated that the expression of PTPN1 would dampen cytokine signaling through attenuating JAK, Tyk2, and STAT5 phosphorylation, thereby inhibiting the expansion and activation of T cells [11]. A similar PTPN1-regulated mechanism was also observed in lymphoid malignancies [29] and Hodgkin lymphoma [30]. It is worth noting that our analysis was interpretable, consistent with the results of molecular experiments to a certain extent. Besides, the results also indicated the feasibility and accuracy of integrating various methods to annotate the enrichment pathway for a single gene. Liver cancer is known to be a multistep development where various signal pathways are altered, such as VEGF receptor signaling, Ras-MAPK signaling and Wnt/*β*-Catenin pathway [31]. Herein, we define an additional pathway in liver cancer: the cytokine-cytokine receptor interaction closely associated with PTPN1.

To further investigate the immune-related role of PTPN1 in liver cancer, we first analyzed the correlation between PTPN1 expression and 47 types of immune checkpoints in the TCGA and ICGC databases. The results showed that most immune checkpoints were increased in the PTPN1 upregulated groups, and most immune checkpoints were positively linked with PTPN1 expression. Among the top 10 PTPN1-correlated immune checkpoints in the TCGA and ICGC databases, the insertion checkpoints including CD28, CD80, and CD86 functioned as T cell regulators [32]. The results suggested that PTPN1 might have a similar expression pattern and immune response effect on T cells as these checkpoints. Next, the TIMER online tools explored six types of immune infiltration cells (B cell, CD4+ T cell, CD8+ T cell, macrophage, neutrophil, and dendritic cell) in liver cancer. The correlation between gene markers of nine immune cells (B cell, T cell, CD4+ T cell, CD8+ T cell, Treg cell, Th cell, macrophage, dendritic cell, and NK cell) and PTPN1 was also analyzed. The results showed a close positive association between PTPN1 and B cell, T cell, CD4+ T cell, CD8+ T cell, Treg cell, macrophage, and dendritic cell. As expected, the infiltration levels or gene markers of distinct T cell subtypes were correlated with PTPN1 expression, further indicating the relationship between PTPN1 and T cell function. Besides, previous research has illustrated the essential role of PTPN1 for T cell activation in mice [33], which undoubtedly supported our conclusion.

Single-cell RNA-seq analysis was further implemented to identify the PTPN1-expressed cell type in the complex tumor microenvironment of liver cancer, which also served as a verification for the above immune analysis. The characteristic profile of liver cancer was obtained through feature reduction, cell clustering, and cell type annotation on GSE146115. Tumor cells, B cells, T cells, macrophages, and NK cells were identified in GSE146115, and PTPN1 was enriched in T cells. Subsequently, we analyzed the T cell population and classified it into CD4+ and CD8+ T cells, and they were PTPN1-expressed cells. Considering that the expression of PTPN1 and T cell markers may be affected by the different cell states, pseudotime analysis on these T cells was then conducted, and it was found that three distinct differentiation states existed. The coexpression pattern of PTPN1 and T cell exhaustion-related genes (CD27, CTLA4, and LAG3) was observed, independent of cell states. The enrichment of PTPN1 in T cells was then validated with the GSE98638 dataset. Using the TISCH online tool, we obtained a comprehensive landscape of T cells infiltrating liver cancer. It was also found that PTPN1 was highly expressed in T cells, including conventional CD4+ T cells, CD8+ T cells, regulatory T cells, exhausted CD8+ T cells, and proliferating T cells. Thus, it might be speculated that PTPN1 was elevated in liver cancer infiltrating T cells and appeared to be associated with T cell exhaustion.

The present findings provided insights into understanding the role of PTP1B in liver cancer, but some limitations still existed. First, we explored mRNA expression levels of PTP1B instead of protein levels because there is a lack of protein omics data matched with adequate clinical information for integrated analysis, just like many bioinformatics analyses on a single gene [13–15, 34, 35]. In this research, the immunohistochemical staining results from the HPA database were used to verify the protein expression level of PTPN1. Second, we speculated that PTPN1 might regulate T cell responses mainly through cytokine-cytokine receptor interaction by pathway enrichment analysis. However, the specific mechanism of how PTPN1 regulates and affects the function of T cell needs more profound molecular research. Third, our analysis was performed using public databases and bioinformatics methods. Therefore, further *in vitro* and *in vivo* research on PTPN1 are needed to verify these computational results, which will make the results more convincing and advance this work.

## 5. Conclusions

Our study showed that the expression of PTPN1 was increased in advanced liver cancer, leading to a worse prognosis for patients. Moreover, PTPN1 was closely associated with some immune response pathways, immune checkpoints, and immune infiltration cells. Notably, T cells were enriched in PTPN1, some of which showed exhaustion characteristics, indicating the inhibition effect of PTPN1 on T cell function. Taken together, these results indicated that PTPN1 (PTP1B) is a promising immunotherapy target associated with T cell function for liver cancer.

## Data Availability

The mRNA expression profiles of liver cancer and corresponding clinical data were obtained from the TCGA database (https://portal.gdc.cancer.gov/) and ICGC database (https://dcc.icgc.org/). Two GEO datasets (GSE146115 and GSE98638) were obtained from the GEO database (https://www.ncbi.nlm.nih.gov/gds/?term=) for single-cell RNA-seq analysis.

## Supplementary Materials

Supplementary Materials
*Supplement Figures.* Supplement Figure S1: study flowchart. Supplement Figure S2: quality control of the single-cellRNA-seq data and PC selection. (a) Data quality control and standardization. (b) Positive correlation between the count of genes and the gene expression levels (R = 0.79). (c) Confirmation of 2000 hypervariable genes for PCA analysis. (d) PC selection (all *p* < 0.001). Supplement Figure S3: cell type annotation by specific gene markers and cell trajectory analysis. (a) UMAP diagram showed 13 cell clusters in GSE146115. (b) Violin diagrams of differential gene marker expression in 13 clusters. (c) UMAP diagram showed 3 clusters of T cell population in GSE146115. (d) Violin diagrams of differential T cell gene marker expression in 3 clusters. (e) Differentiation trajectory of T cells on clusters, cell type, and pseudotime. *Supplement Tables.* Supplement Table 1: gene list for Spearman's correlation analysis. Supplement Table 2: the significantly positive PTPN1-correlated genes.

## References

[1] Sung H., Ferlay J., Siegel R. L. (2021). Global cancer statistics 2020: GLOBOCAN estimates of incidence and mortality worldwide for 36 cancers in 185 countries. *CA: A Cancer Journal for Clinicians*.

[2] Xu F., Jin T., Zhu Y., Dai C. (2018). Immune checkpoint therapy in liver cancer. *Journal of Experimental and Clinical Cancer Research*.

[3] Chen Z., Xie H., Hu M. (2020). Recent progress in treatment of hepatocellular carcinoma. *Am J Cancer Res*.

[4] Giraud J., Chalopin D., Blanc J. F., Saleh M. (2021). Hepatocellular carcinoma immune landscape and the potential of immunotherapies. *Frontiers in Immunology*.

[5] Anwanwan D., Singh S. K., Singh S., Saikam V., Singh R. (2020). Challenges in liver cancer and possible treatment approaches. *Biochimica et Biophysica Acta (BBA) - Reviews on Cancer*.

[6] Llovet J. M., Castet F., Heikenwalder M. (2022). Immunotherapies for hepatocellular carcinoma. *Nature Reviews Clinical Oncology*.

[7] Zhu X. D., Huang C., Shen Y. H. (2021). Downstaging and resection of initially unresectable hepatocellular carcinoma with tyrosine kinase inhibitor and anti-PD-1 antibody combinations. *Liver Cancer*.

[8] Peng W., Chen J. Q., Liu C. (2016). Loss of PTEN promotes resistance to T cell-mediated immunotherapy. *Cancer Discovery*.

[9] Guo J., Tang Q. (2021). Recent updates on chimeric antigen receptor T cell therapy for hepatocellular carcinoma. *Cancer Gene Therapy*.

[10] Kumar A., Rana D., Rana R., Bhatia R. (2020). Protein tyrosine phosphatase (PTP1B): a promising drug target against life-threatening ailments. *Current Molecular Pharmacology*.

[11] Wiede F., Lu K. H., Du X. (2022). PTP1B is an intracellular checkpoint that limits T-cell and CAR T-cell antitumor immunity. *Cancer Discovery*.

[12] Villamar-Cruz O., Loza-Mejía M. A., Arias-Romero L. E., Camacho-Arroyo I. (2021). Recent advances in PTP1B signaling in metabolism and cancer. *Bioscience Reports*.

[13] Fan Y., Liu B., Chen F. (2021). Hepcidin upregulation in lung cancer: a potential therapeutic target associated with immune infiltration. *Frontiers in Immunology*.

[14] Huang Z., Yang J., Qiu W. (2022). HAUS5 is A potential prognostic biomarker with functional significance in breast cancer. *Frontiers in Oncology*.

[15] Yu L., Ding Y., Wan T., Deng T., Huang H., Liu J. (2021). Significance of CD47 and its association with tumor immune microenvironment heterogeneity in ovarian cancer. *Frontiers in Immunology*.

[16] Su X., Zhao L., Shi Y. (2021). Clonal evolution in liver cancer at single-cell and single-variant resolution. *Journal of Hematology and Oncology*.

[17] Zheng C., Zheng L., Yoo J. K. (2017). Landscape of infiltrating T cells in liver cancer revealed by single-cell sequencing. *Cell*.

[18] Li T., Fan J., Wang B. (2017). TIMER: a web server for comprehensive analysis of tumor-infiltrating immune cells. *Cancer Research*.

[19] Chandrashekar D. S., Bashel B., Balasubramanya S. A. H. (2017). UALCAN: a portal for facilitating tumor subgroup gene expression and survival analyses. *Neoplasia*.

[20] Qi Y., Xia Y., Lin Z. (2020). Tumor-infiltrating CD39(+) CD8(+) T cells determine poor prognosis and immune evasion in clear cell renal cell carcinoma patients. *Cancer Immunology, Immunotherapy*.

[21] Bhattacharya S., Dunn P., Thomas C. G. (2018). ImmPort, toward repurposing of open access immunological assay data for translational and clinical research. *Scientific Data*.

[22] Zhang X., Lan Y., Xu J. (2019). CellMarker: a manually curated resource of cell markers in human and mouse. *Nucleic Acids Research*.

[23] Sun D., Wang J., Han Y. (2021). TISCH: a comprehensive web resource enabling interactive single-cell transcriptome visualization of tumor microenvironment. *Nucleic Acids Research*.

[24] Xu X., Tao Y., Niu Y. (2019). miR-125a-5p inhibits tumorigenesis in hepatocellular carcinoma. *Aging (Albany NY)*.

[25] Yang Q., Zhang L., Zhong Y., Lai L., Li X. (2019). miR-206 inhibits cell proliferation, invasion, and migration by down-regulating PTP1B in hepatocellular carcinoma. *Bioscience Reports*.

[26] Subramanian A., Tamayo P., Mootha V. K. (2005). Gene set enrichment analysis: a knowledge-based approach for interpreting genome-wide expression profiles. *Proceedings of the National Academy of Sciences of the United States of America*.

[27] Guimond M., Fry T. J., Mackall C. L. (2005). Cytokine signals in T-cell homeostasis. *Journal of Immunotherapy*.

[28] Dong C. (2021). Cytokine regulation and function in T cells. *Annual Review of Immunology*.

[29] Pike K. A., Tremblay M. L. T. C.-P. T. P. (2016). TC-PTP and PTP1B: regulating JAK–STAT signaling, controlling lymphoid malignancies. *Cytokine*.

[30] Zahn M., Kaluszniak B., Möller P., Marienfeld R. (2021). The PTP1B mutant PTP1B∆2-4 is a positive regulator of the JAK/STAT signalling pathway in Hodgkin lymphoma. *Carcinogenesis*.

[31] Bruix J., Han K. H., Gores G., Llovet J. M., Mazzaferro V. (2015). Liver cancer: approaching a personalized care. *Journal of Hepatology*.

[32] Lane P. (1997). Regulation of T and B cell responses by modulating interactions between CD28/CTLA4 and their ligands, CD80 and CD86. *Annals of the New York Academy of Sciences*.

[33] Martin-Granados C., Prescott A. R., Le Sommer S. (2015). A key role for PTP1B in dendritic cell maturation, migration, and T cell activation. *Journal of Molecular Cell Biology*.

[34] Di W., Fan W., Wu F. (2022). Clinical characterization and immunosuppressive regulation of CD161 (KLRB1) in glioma through 916 samples. *Cancer Science*.

[35] Cui X., Zhang X., Liu M. (2020). A pan-cancer analysis of the oncogenic role of staphylococcal nuclease domain-containing protein 1 (SND1) in human tumors. *Genomics*.

