# Supplementary figures and images for: Comprehensive Bioinformatics Analysis Reveals PTPN1 (PTP1B) Is a Promising Immunotherapy Target Associated with T Cell Function for Liver Cancer
Source: J Healthc Eng. 2023 Jan 27;2023:1533794. doi: 10.1155/2023/1533794 (PMC9897930; doi:10.1155/2023/1533794)

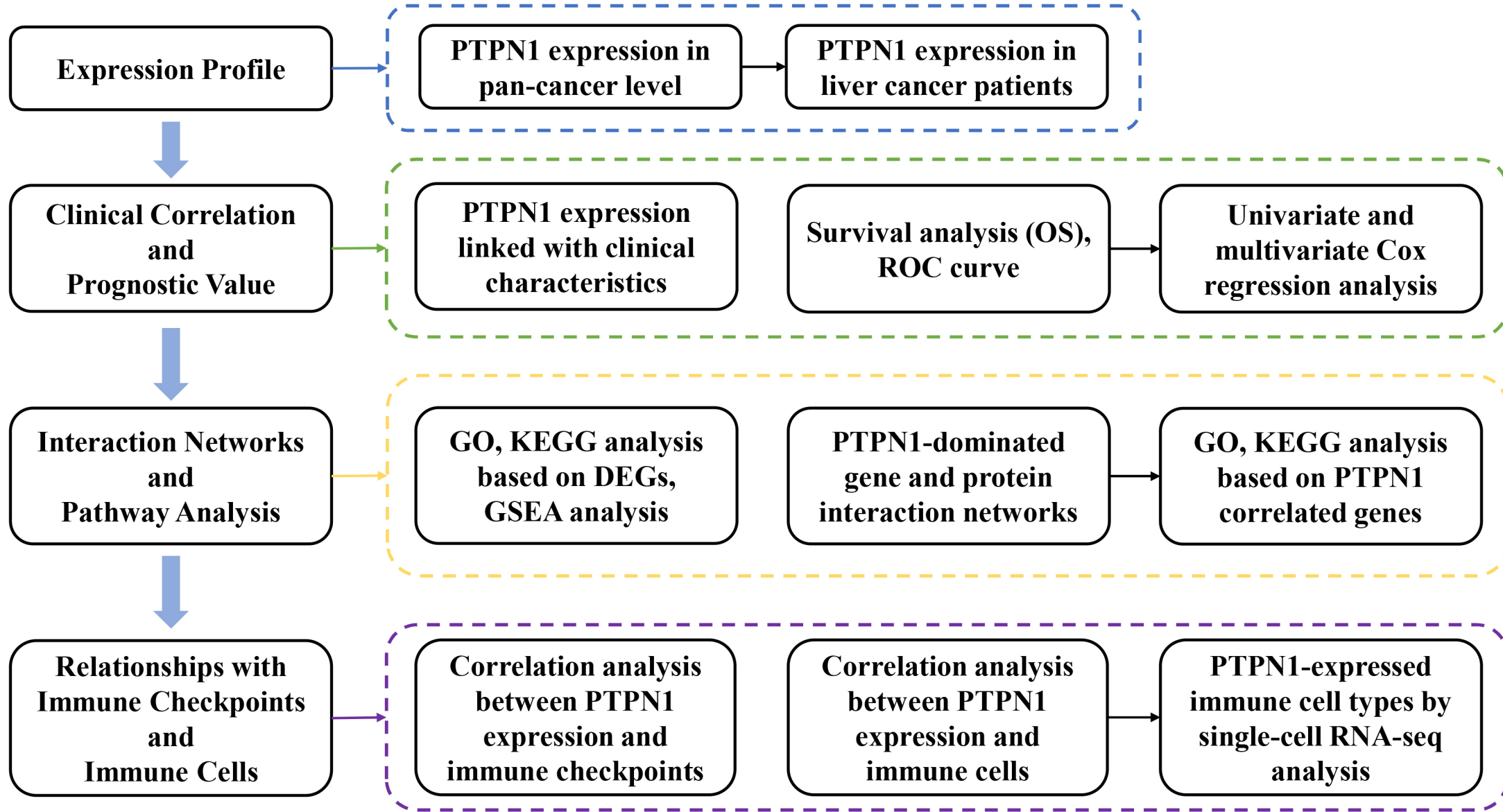

Supplement: Supplementary Materials — Supplement Figures. Supplement Figure S1: study flowchart. Supplement Figure S2: quality control of the single-cellRNA-seq data and PC selection. (a) Data quality control and standardization. (b) Positive correlation between the count of genes and the gene expression levels (R = 0.79). (c) Confirmation of 2000 hypervariable genes for PCA analysis. (d) PC selection (all p < 0.001). Supplement Figure S3: cell type annotation by specific gene markers and cell trajectory analysis. (a) UMAP diagram showed 13 cell clusters in GSE146115. (b) Violin diagrams of differential gene marker expression in 13 clusters. (c) UMAP diagram showed 3 clusters of T cell population in GSE146115. (d) Violin diagrams of differential T cell gene marker expression in 3 clusters. (e) Differentiation trajectory of T cells on clusters, cell type, and pseudotime. Supplement Tables. Supplement Table 1: gene list for Spearman's correlation analysis. Supplement Table 2: the significantly positive PTPN1-correlated genes. [file 1533794.f1.zip › Supplement Figure S1.pdf]

**a**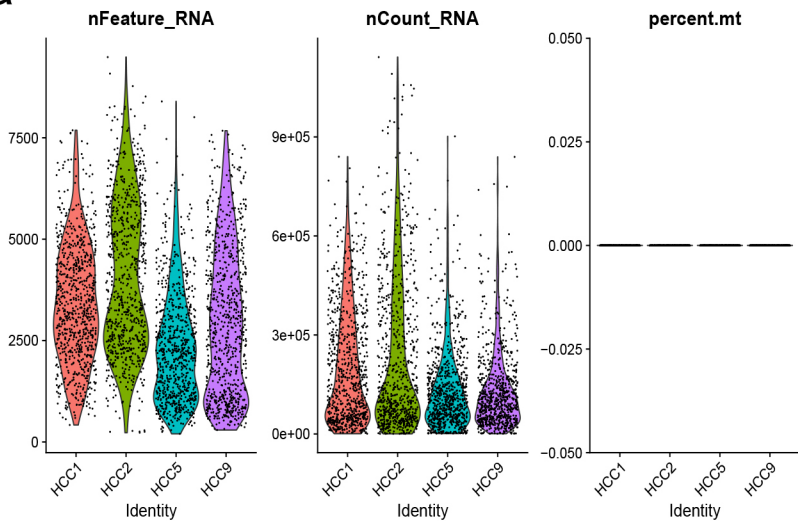**b**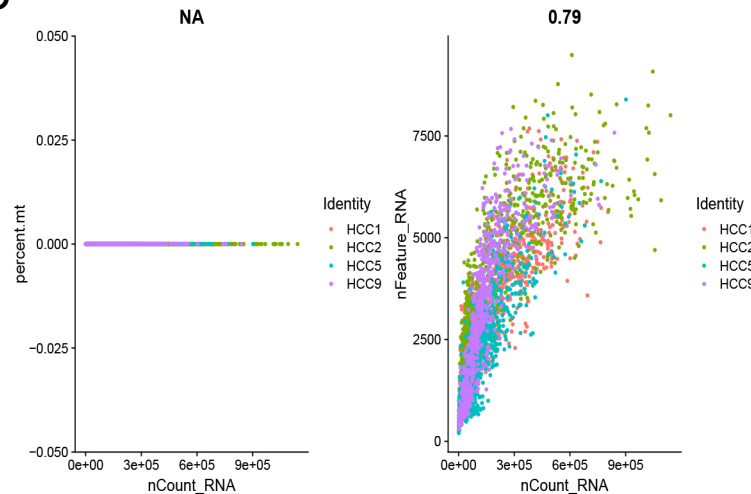**c**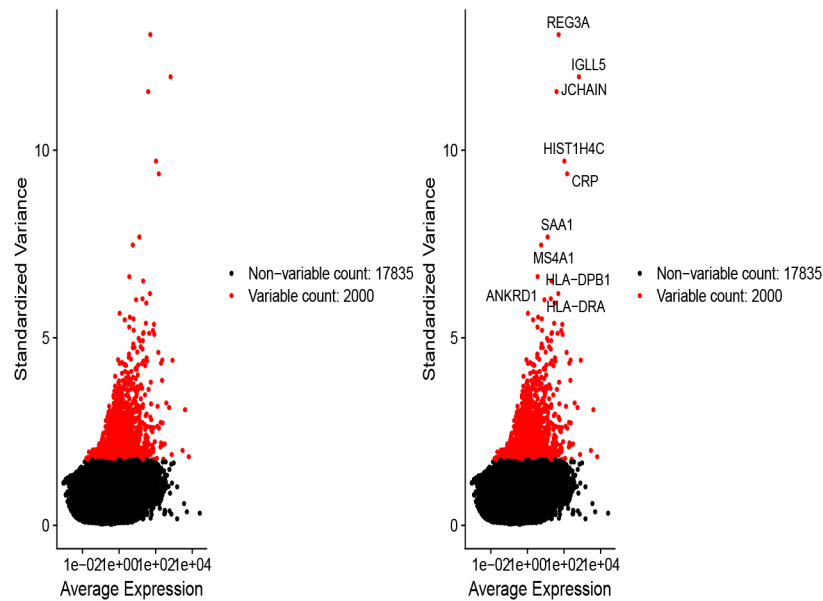**d**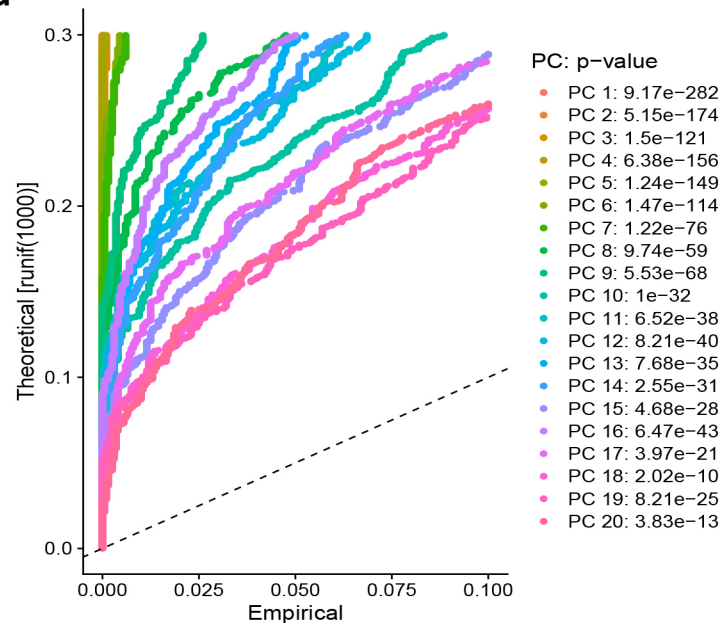

Supplement: Supplementary Materials — Supplement Figures. Supplement Figure S1: study flowchart. Supplement Figure S2: quality control of the single-cellRNA-seq data and PC selection. (a) Data quality control and standardization. (b) Positive correlation between the count of genes and the gene expression levels (R = 0.79). (c) Confirmation of 2000 hypervariable genes for PCA analysis. (d) PC selection (all p < 0.001). Supplement Figure S3: cell type annotation by specific gene markers and cell trajectory analysis. (a) UMAP diagram showed 13 cell clusters in GSE146115. (b) Violin diagrams of differential gene marker expression in 13 clusters. (c) UMAP diagram showed 3 clusters of T cell population in GSE146115. (d) Violin diagrams of differential T cell gene marker expression in 3 clusters. (e) Differentiation trajectory of T cells on clusters, cell type, and pseudotime. Supplement Tables. Supplement Table 1: gene list for Spearman's correlation analysis. Supplement Table 2: the significantly positive PTPN1-correlated genes. [file 1533794.f1.zip › Supplement Figure S2.pdf]

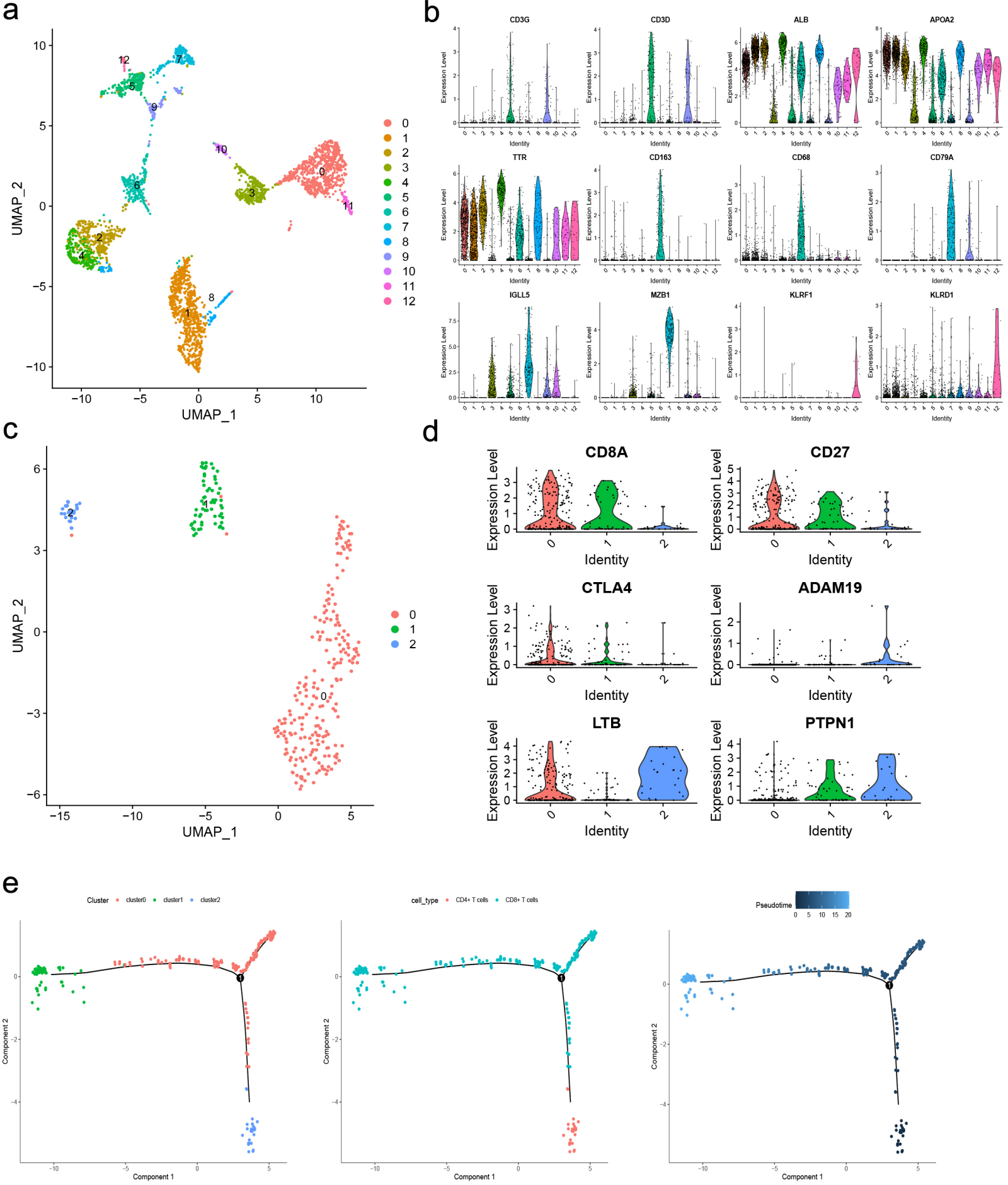

Supplement: Supplementary Materials — Supplement Figures. Supplement Figure S1: study flowchart. Supplement Figure S2: quality control of the single-cellRNA-seq data and PC selection. (a) Data quality control and standardization. (b) Positive correlation between the count of genes and the gene expression levels (R = 0.79). (c) Confirmation of 2000 hypervariable genes for PCA analysis. (d) PC selection (all p < 0.001). Supplement Figure S3: cell type annotation by specific gene markers and cell trajectory analysis. (a) UMAP diagram showed 13 cell clusters in GSE146115. (b) Violin diagrams of differential gene marker expression in 13 clusters. (c) UMAP diagram showed 3 clusters of T cell population in GSE146115. (d) Violin diagrams of differential T cell gene marker expression in 3 clusters. (e) Differentiation trajectory of T cells on clusters, cell type, and pseudotime. Supplement Tables. Supplement Table 1: gene list for Spearman's correlation analysis. Supplement Table 2: the significantly positive PTPN1-correlated genes. [file 1533794.f1.zip › Supplement Figure S3.pdf]
